# Understanding Genetic Screening: Harnessing Health Information to Prevent Disease Risks

**DOI:** 10.7150/ijms.101219

**Published:** 2025-01-21

**Authors:** Chung-Lin Lee, Chih-Kuang Chuang, Huei-Ching Chiu, Ya-Hui Chang, Yuan-Rong Tu, Yun-Ting Lo, Hsiang-Yu Lin, Shuan-Pei Lin

**Affiliations:** 1 Department of Pediatrics, MacKay Memorial Hospital, Taipei, Taiwan.; 2 Institute of Clinical Medicine, National Yang-Ming Chiao-Tung University, Taipei, Taiwan.; 3 International Rare Disease Center, MacKay Memorial Hospital, Taipei, Taiwan.; 4 Department of Medicine, Mackay Medical College, New Taipei City, Taiwan.; 5 Mackay Junior College of Medicine, Nursing and Management, Taipei, Taiwan.; 6 Division of Genetics and Metabolism, Department of Medical Research, MacKay Memorial Hospital, Taipei, Taiwan.; 7 College of Medicine, Fu-Jen Catholic University, Taipei, Taiwan.; 8 Department of Medical Research, China Medical University Hospital, China Medical University, Taichung, Taiwan.; 9 Department of Infant and Child Care, National Taipei University of Nursing and Health Sciences, Taipei, Taiwan.

**Keywords:** Genetic Screening, Health Management, Disease Prevention, Personalized Medicine, Genetic Disorders

## Abstract

Genetic screening analyzes an individual's genetic information to assess disease risk and provide personalized health recommendations. This article introduces the public to genetic screening, explaining its definition, principles, history, and common types, including prenatal, newborn, adult disease risk, cancer, and pharmacogenetic screening. It elaborates on the benefits of genetic screening, such as early risk detection, personalized prevention, family risk assessment, and reproductive decision-making. The article also notes limitations, including result interpretation uncertainty, psychological and ethical issues, and privacy and discrimination risks. It provides advice on selecting suitable screening, consulting professionals, choosing reliable institutions, and understanding screening purposes and limitations. Finally, it discusses applying screening results through lifestyle adjustments, regular check-ups, and preventive treatments. By comprehensively introducing genetic screening, the article aims to raise public awareness and encourage utilizing this technology to prevent disease and maintain health.

## I. Introduction

### A. The Importance of Genetic Screening

Genetic screening occupies an increasingly important position in modern medicine and personal health management. The following points illustrate the significant implications of genetic screening:

1. Early Identification of Health Risks: Genetic screening can detect an individual's risk of developing certain diseases before clinical symptoms appear. This provides valuable time for early intervention and prevention, which helps improve disease prognosis and reduce the disease burden on individuals and society 1.

2. Personalized Medicine and Prevention Strategies: The results of genetic screening provide key information for medical professionals, helping them formulate medical and prevention plans tailored to individual characteristics. This precision medicine approach can improve treatment efficacy, reduce adverse reactions, and optimize the implementation of preventive measures 2.

3. Family Risk Assessment: Many diseases are hereditary, and the results of genetic screening are of great significance for the health management of both the person being screened and their family members. After identifying the risk of familial diseases, family members can take appropriate preventive and monitoring measures to detect and treat potential health problems early 3.

4. Reference for Reproductive Decisions: Genetic screening can help prospective parents understand their risk of carrying genetic diseases, providing an important reference for reproductive decisions. This helps reduce the incidence of genetic diseases in offspring and improve the health level of newborns 4.

5. Promoting Scientific Research and Medical Progress: The large amount of data generated by genetic screening provides valuable resources for scientific research. By analyzing these data, researchers can gain in-depth understanding of the genetic basis of diseases, discover new disease markers and therapeutic targets, and promote medical progress and innovation 5.

In summary, genetic screening plays an important role in disease prevention, personalized medicine, family health management, reproductive decision-making, and scientific research. As technology advances and applications become more widespread, genetic screening is expected to play an even greater role in protecting human health and improving quality of life.

### B. The Main Purpose and Content Overview of This Article

This article aims to comprehensively introduce the general public to knowledge related to genetic screening, enhancing people's awareness and attention to this advanced technology. Through easy-to-understand explanations and analysis, the article hopes to help readers understand the importance of genetic screening in personal health management and disease prevention, and provide practical guidance and suggestions.

The content of the article covers various aspects of genetic screening. First, the article will explain the basic concepts of genetic screening, including its definition, principles, and development history, providing readers with the necessary theoretical foundation 6. Next, the article will introduce several common types of genetic screening in detail, such as prenatal screening, newborn screening, adult disease risk screening, etc., helping readers fully understand the application fields of genetic screening 7,8.

When elaborating on the benefits of genetic screening, the article will emphasize its important value in early detection of health risks, formulation of personalized prevention strategies, assessment of risks for family members, and assisting reproductive decision-making 9. At the same time, in order to make the content objective and comprehensive, the article will also analyze the limitations of genetic screening, such as the uncertainty of interpreting results, potential psychological and ethical issues, and challenges in privacy protection 10.

In addition, the article will provide readers with practical advice and guidance, including how to choose reliable genetic screening institutions and how to interpret and apply screening results 11. Through this content, the article hopes to help the public make informed decisions and fully realize the positive role of genetic screening.

In summary, through comprehensive, in-depth, and practical introductions, this article will enhance the public's cognitive level of genetic screening, encouraging more people to actively learn about and utilize this advanced technology to provide strong support and protection for the health management of individuals and families.

The originality of this article is reflected in the following aspects: First, we comprehensively and systematically review various aspects of genetic screening from a public health and healthcare policy perspective, providing a holistic view. Second, we focus particularly on the application of genetic screening in personalized health management, which is a rapidly developing emerging field. Finally, we delve into the ethical and social challenges faced by genetic screening, providing important references for future policymaking.

## II. What is Genetic Screening?

### A. Definition of Genetic Screening

Genetic screening utilizes molecular biology techniques to detect specific genetic variants in an individual's DNA that may be associated with genetic diseases or disease risk 12. Medical professionals can assess an individual's risk of developing certain diseases or diagnose genetic diseases by analyzing these results 13.

Specifically, genetic screening typically includes the following steps: First, DNA is extracted from the individual's blood, saliva, or other tissue samples. Next, various molecular biology techniques, such as polymerase chain reaction (PCR) and DNA sequencing, are used to analyze the target gene regions and detect specific genetic variants. Finally, professionals assess the individual's health risks or diagnosis based on the test results and other clinical information 14.

Genetic screening has a wide range of applications and purposes, including newborn screening, prenatal screening, adult disease risk assessment, pharmacogenetic testing, etc. 15. These screenings help with early detection of genetic diseases, prediction of disease risks, guidance for treatment plan formulation, and assistance with reproductive decisions 16.

In general, genetic screening is a powerful tool for disease prevention, diagnosis, and treatment by analyzing an individual's genetic information. As genomic knowledge deepens and technology advances, genetic screening is expected to play an increasingly important role in personalized medicine and public health 17.

### B. Principles of Genetic Screening

The principles of genetic screening are based on the diversity of the human genome. Our genome contains many variants, including single nucleotide polymorphisms (SNPs), copy number variations (CNVs), insertions and deletions (Indels), etc. 18. Certain variants may affect gene function and thus be associated with specific diseases or disease risks 19. Genetic screening assesses an individual's risk of developing certain genetic diseases or makes a diagnosis by detecting these key variants.

The specific principles of genetic screening involve a variety of molecular biology techniques. Currently, the most commonly used methods are polymerase chain reaction (PCR) and DNA sequencing 20. PCR is a rapid and sensitive DNA amplification technique that selectively amplifies the target gene region by using specific primers and detects variants within it. DNA sequencing is a more comprehensive and high-throughput method that determines the exact sequence of DNA fragments and identifies various variants within them 21.

In recent years, with the rapid development of genomics, new screening technologies have emerged. For example, microarray technology can simultaneously detect thousands of SNPs in a single experiment, greatly improving screening efficiency 22. Another important advancement is the application of next-generation sequencing (NGS) technology, which can quickly and economically sequence the entire genome or exome, providing a powerful tool for more comprehensive and in-depth analysis of genetic variants 23.

In summary, the principle of genetic screening is to use various molecular biology techniques to detect key variants in an individual's genome that are associated with diseases, thereby assessing disease risk or making a diagnosis. As technology continues to advance and genomic knowledge becomes increasingly rich, genetic screening will undoubtedly play an increasingly important role in personalized medicine and disease prevention.

### C. Mechanisms of Genetic Diseases

Genetic diseases are primarily caused by abnormalities in genes or chromosomes 24. Chromosomal abnormalities, such as the absence of some or all chromosomes, are referred to as aneuploidy 25. Changes or mutations in genes are the root cause of many hereditary diseases 26. For example, Tay-Sachs disease, sickle cell disease, and cystic fibrosis are all caused by mutations in specific genes 27. Often, a child needs to inherit the same defective gene from both parents to be affected. This inheritance pattern is known as autosomal recessive inheritance 28.

However, the mechanisms of genetic diseases are not always so simple. Some genetic diseases may involve the interaction of multiple genes, or the interaction between genes and environmental factors 29. Moreover, epigenetic modifications can also affect gene expression, thereby influencing the occurrence and development of diseases 30.

Understanding these mechanisms of genetic diseases is crucial for genetic screening. It not only helps us determine screening targets but also aids in correctly interpreting screening results and providing more accurate genetic counseling for patients and their families 31.

### D. Technical Methods of Genetic Screening

1. Polymerase Chain Reaction (PCR):

- Principle: Exponential amplification of target DNA fragments using specific primers and DNA polymerase 32.

- Application: Commonly used for detecting specific gene mutations, such as CFTR gene mutations in cystic fibrosis 33.

- Advantages: High sensitivity, strong specificity, relatively simple operation 34.

- Limitations: Can only detect a limited number of gene loci at a time 34.

2. DNA Sequencing:

a. Sanger Sequencing:

- Principle: Determines DNA sequence using the chain-termination method 35.

- Application: Suitable for mutation detection and verification of known genes 36.

- Advantages: High accuracy, gold standard for many clinical diagnoses 36.

- Limitations: Relatively low throughput, higher cost 36.

b. Next-Generation Sequencing (NGS):

- Principle: Massive parallel sequencing, simultaneously sequencing millions of DNA fragments 37.

- Application: Whole exome sequencing, whole genome sequencing, targeted gene panel sequencing, etc. 38.

- Advantages: High throughput, cost-effective, can detect unknown mutations 38.

- Limitations: Complex data analysis, high requirements for bioinformatics 38.

3. Microarray Technology:

- Principle: Uses probes fixed on chips to detect specific sequences in samples 39.

- Application: Single Nucleotide Polymorphism (SNP) detection, Copy Number Variation (CNV) analysis 40.

- Advantages: Can simultaneously detect a large number of loci, relatively low cost 40.

- Limitations: Cannot detect new or rare variants 40.

4. Digital PCR:

- Principle: Divides samples into thousands of micro-reaction volumes, enabling absolute quantification 41.

- Application: Rare mutation detection, copy number variation analysis 42.

- Advantages: Extremely high sensitivity and precision 42.

- Limitations: Expensive equipment, relatively limited throughput 42.

5. Long-Read Sequencing:

- Principle: Can directly sequence long DNA fragments, such as PacBio and Oxford Nanopore technologies 43.

- Application: Structural variation detection, sequencing of complex regions 44.

- Advantages: Can detect large insertions, deletions, and rearrangements 44.

- Limitations: Relatively low single-base accuracy, higher cost 44.

6. Epigenetic Analysis Methods:

- Principle: Detects epigenetic markers such as DNA methylation and histone modifications 45.

- Application: Methylation-specific PCR, ChIP-seq, etc. 46.

- Advantages: Provides information on gene expression regulation 46.

- Limitations: High sample processing requirements, complex result interpretation 46.

These technical methods each have their advantages and disadvantages. Choosing the appropriate method is crucial for accurate and efficient genetic screening. With the continuous advancement of technology, the accuracy, coverage, and cost-effectiveness of genetic screening are constantly improving 47.

### E. Development History of Genetic Screening (Table 1)

The development history of genetic screening can be traced back to the 1960s. In 1963, American geneticist Robert Guthrie developed a simple bacterial inhibition assay for screening newborns for phenylketonuria, marking the beginning of newborn screening 48. As molecular biology progressed, genetic screening technology continued to evolve.

In the 1970s, the discovery of two major foundational techniques in molecular biology—DNA molecular hybridization and restriction endonucleases—laid the technical foundation for genetic screening 49. In 1977, Frederick Sanger invented DNA sequencing technology, making it possible to directly read DNA sequences, which had a profound impact on the development of genetic screening 50.

In the 1980s, the invention and application of PCR technology brought revolutionary changes to genetic screening. PCR can rapidly and specifically amplify trace amounts of DNA, greatly improving the sensitivity and efficiency of genetic screening 51. During this period, screening for many important genetic diseases was realized, such as sickle cell anemia, thalassemia, etc. 52.

Entering the 21st century, advances in microarray and gene sequencing technologies have further propelled the development of genetic screening. Microarrays can analyze a large number of gene loci simultaneously in a single experiment, making large-scale genetic disease screening and disease risk assessment possible 53. The emergence of next-generation sequencing technologies, such as Illumina sequencing and Ion Torrent sequencing, has greatly increased the throughput of DNA sequencing and significantly reduced costs, laying the foundation for more comprehensive and economical genetic screening 54,55.

Overall, genetic screening technology has made great strides over the past half century. From initial newborn screening to now covering multiple fields such as prenatal diagnosis, genetic disease testing, and tumor gene testing, genetic screening has become an important tool in modern medicine. As genomic research continues to deepen and technology evolves, genetic screening is bound to play an increasingly important role in personalized medicine.

## III. Types of Genetic Screening (Table 2)

### A. Prenatal Genetic Screening

Prenatal genetic screening is an examination performed before the birth of a fetus to identify whether the fetus has a risk of certain genetic diseases or chromosomal abnormalities. This screening is usually performed at specific times during pregnancy and can help prospective parents understand the health status of the fetus and provide important information for subsequent medical decisions 56.

Currently, common prenatal genetic screening methods include maternal blood Down syndrome screening, non-invasive prenatal genetic testing (NIPT), and invasive prenatal diagnosis (such as chorionic villus sampling and amniocentesis) 57. Maternal blood Down syndrome screening assesses the risk of the fetus having Down syndrome by measuring specific markers in maternal blood; NIPT screens for common chromosomal abnormalities in the fetus by analyzing free fetal DNA fragments in maternal blood; invasive prenatal diagnosis requires obtaining fetal tissue samples and can more accurately diagnose genetic diseases and chromosomal abnormalities 58,59.

An important advantage of prenatal genetic screening is that it can detect fetal health problems as early as possible, giving families more time to prepare or, in some cases, consider termination of pregnancy 60. However, these screenings also have some limitations, such as the possibility of false positive and false negative results, and the inability to detect certain rare diseases 61. In addition, screening results may have psychological and ethical impacts on prospective parents, so genetic counseling and support from medical professionals are needed 62.

In general, prenatal genetic screening provides an important tool for assessing fetal health. As technology advances and new screening methods emerge, prenatal genetic screening is expected to play an increasingly important role in prenatal care. At the same time, medical institutions and all sectors of society should also strengthen education and support for prospective parents to help them correctly understand and utilize the results of prenatal genetic screening.

### B. Newborn Genetic Screening

Newborn genetic screening is an important public health measure aimed at early detection and treatment of certain genetic diseases that may endanger infant health. This screening is usually performed shortly after the infant's birth by testing the infant's blood or other tissue samples to screen for a range of genetic diseases 63.

The content of newborn genetic screening varies by country and region, but usually includes some relatively common diseases that can be effectively treated, such as phenylketonuria, congenital hypothyroidism, etc. 64. Among them, phenylketonuria is a type of amino acid metabolism abnormality that, if not treated in time, can lead to impaired intellectual development in infants 65; congenital hypothyroidism affects infant growth and development and intelligence 66.

One major advantage of newborn genetic screening is that it can intervene before diseases cause irreversible damage. Through timely diagnosis and appropriate treatment, many adverse consequences of genetic diseases can be prevented or mitigated 64. For example, infants with phenylketonuria can develop normally if they receive a low-phenylalanine diet 65; infants with congenital hypothyroidism can also avoid intellectual impairment if they receive thyroid hormone supplementation in time 66.

However, newborn genetic screening also has some limitations and ethical issues. The interpretation of screening results is sometimes difficult and may bring anxiety and uncertainty to families 67. In addition, the significance of screening results for certain genetic diseases that lack effective treatment is also questionable 68. Therefore, the selection of newborn genetic screening items should be carefully weighed, considering factors such as disease severity, treatment possibility, and cost-effectiveness of screening.

In general, newborn genetic screening, as an important public health measure, plays a crucial role in the prevention and treatment of genetic diseases. As screening technology advances and new treatment methods emerge, the scope and effectiveness of newborn genetic screening is expected to further improve. At the same time, we should also pay attention to the ethical and social issues that newborn screening may bring, ensuring that each family can make informed choices and receive necessary support and help.

### C. Adult Disease Risk Genetic Screening

Adult disease risk genetic screening aims to identify an individual's genetic risk of developing certain common diseases (such as cardiovascular disease, type 2 diabetes, etc.). By analyzing specific genetic variants, these screenings can assess an individual's disease susceptibility and help them take preventive measures 69.

Some well-known adult disease risk genetic screening items include BRCA1/2 genes (breast and ovarian cancer risk) 70, APOE gene (Alzheimer's disease risk) 71, etc. However, the interpretation of these screening results is often complex, and many genetic variants can only explain a small portion of disease risk 72. Therefore, the results of adult disease risk genetic screening should be comprehensively evaluated in conjunction with other factors such as family history and lifestyle, and appropriate genetic counseling should be provided by professionals 73.

In addition, adult disease risk genetic screening also involves some ethical and social issues, such as the psychological impact screening results may have on individuals, or lead to discrimination and privacy leakage, etc. 74. Therefore, screening institutions should strictly abide by the principle of informed consent, protect the rights and interests of those being screened, and provide them with necessary support and protection.

### D. Cancer Genetic Screening

Cancer genetic screening mainly targets certain hereditary tumor syndromes, such as hereditary breast and ovarian cancer syndrome, Lynch syndrome, etc. These diseases are associated with specific gene mutations, and family members carrying the mutations have a higher risk 75.

Currently, multiple cancer genetic screenings have been applied clinically, such as BRCA1/2 gene testing (breast and ovarian cancer), MLH1/MSH2 gene testing (colorectal cancer), etc. 76. For high-risk populations, cancer genetic screening can help them detect tumors early or take preventive measures (such as prophylactic surgery), thereby improving prognosis 77.

However, cancer genetic screening also has some limitations. The pathogenic mechanisms of many hereditary tumors have not been fully elucidated, and there is uncertainty in the interpretation of screening results 78. In addition, positive results may bring a heavy psychological burden to those being screened, and some preventive measures (such as prophylactic mastectomy) may also have a negative impact on quality of life 79. Therefore, cancer genetic screening should be based on evidence-based medicine and supplemented by professional genetic counseling and psychological support.

### E. Pharmacogenetic Screening

Pharmacogenetic screening utilizes an individual's genetic information for drug therapy, with the aim of achieving personalized medication and reducing adverse reactions. This screening mainly targets genes related to drug metabolism enzymes, drug targets, and drug transport proteins 80.

Some common pharmacogenetic screening items include CYP2C19 genotype (clopidogrel metabolism) 81, VKORC1 and CYP2C9 genotypes (warfarin dosage) 82, TPMT genotype (azathioprine toxicity) 83, etc. With the help of these screenings, clinicians can adjust drug dosages or choose alternative drugs based on the patient's genotype, thereby improving efficacy, reducing the risk of adverse reactions 84.

Although pharmacogenetic screening has broad application prospects, it still faces some challenges at present. The clinical significance of many drug-related genes is still unclear, and there is a lack of unified standards for the interpretation and application of screening results 85. In addition, genotype is only one of many factors that affect drug response; others such as age, concomitant medications, and liver and kidney function also need to be taken into consideration 86. In the future, pharmacogenetic screening still requires more clinical validation and guideline development to be better utilized in clinical practice.

## IV. Benefits of Genetic Screening

### A. Early Detection of Potential Health Risks

One of the most significant benefits of genetic screening is that it can identify potential health risks early, even before clinical symptoms appear. This provides valuable time for taking preventive measures and early treatment, which helps improve disease prognosis and quality of life 87.

Taking hereditary cancers as an example, individuals carrying pathogenic mutations have a significantly increased risk of developing cancer. By identifying this risk early through genetic screening, personalized screening and prevention strategies can be developed, such as increasing screening frequency, taking preventive medications, or considering prophylactic surgery in specific cases, thereby greatly reducing the incidence and mortality of cancer 88.

For some common adult diseases such as cardiovascular disease and type 2 diabetes, early identification of genetic risks is also of great significance. This can help high-risk individuals adjust their lifestyles in a timely manner, control related risk factors, and delay or prevent the occurrence and progression of diseases 89.

### B. Personalized Medicine and Prevention Strategies

Another major advantage of genetic screening is that it can provide personalized medical and prevention strategies. The traditional "one-size-fits-all" treatment model ignores individual differences among patients, while genetic screening can "tailor" medical plans according to each person's unique genetic background and disease risk 90.

In the field of drug therapy, pharmacogenetic screening has shown great application potential. By testing genetic variants related to drug metabolism, transport, and targets, clinicians can optimize drug selection and dosage, achieve precision medication, improve efficacy, and reduce the risk of adverse reactions 91. Some countries and regions have incorporated pharmacogenetic testing into routine clinical practice, such as the DPWG guidelines in the Netherlands and the CPIC guidelines in the United States 92.

In the field of disease prevention, genetic screening results can be used to develop personalized prevention strategies. For high-risk individuals, more aggressive preventive measures can be taken, such as increasing screening frequency and taking preventive medications; for low-risk individuals, unnecessary treatments can be avoided, reducing overmedication 93. This risk-based prevention strategy can not only improve prevention efficiency but also save medical resources and reduce the medical burden.

### C. Risk Assessment for Family Members

Many genetic diseases are hereditary, and direct relatives of patients often face a higher risk of developing the disease. Genetic screening can not only identify patients who have already developed the disease but also assess the risk of family members carrying pathogenic variants 94. This provides an entry point for conducting family investigations and cascade screening.

Family investigations help identify high-risk families and conduct targeted screening and interventions for other members of the family, with the goal of detecting potential patients as early as possible 95. This is especially important for some incurable genetic diseases in late stages (such as Huntington's disease), allowing family members to be aware of their risks and make life and reproductive plans early 96.

Cascade screening takes known mutation carriers as a starting point and conducts targeted mutation testing on their blood relatives. This family-based screening strategy can maximize the identification of high-risk individuals and implement prevention and management for them, thereby interrupting the transmission of genetic diseases 97. Some countries have incorporated cascade screening into their genetic disease prevention and control systems, such as the Netherlands' familial hypercholesterolemia (FH) cascade screening program 98.

### D. Reference for Reproductive Decisions

An important application of genetic screening is to provide a reference for reproductive decisions. Prospective parents can understand whether they carry pathogenic variants of certain recessive genetic diseases through carrier screening, thereby assessing the risk of their offspring developing these diseases 99. This helps make informed reproductive choices.

For high-risk couples, some measures can be taken to reduce the risk of their offspring developing the disease, such as preimplantation genetic diagnosis (PGD) 100. PGD technology allows genetic analysis of embryos before implantation, screening out healthy embryos that do not carry pathogenic variants, thereby preventing genetic diseases from the source 101.

Although reproductive decision-making remains a complex and sensitive topic, genetic screening undoubtedly provides prospective parents with more information and choices. Through genetic counseling, professionals can help prospective parents weigh the pros and cons and make the decision that best suits their family 102.

## V. Limitations of Genetic Screening

### A. Interpretation and Uncertainty of Screening Results

Although genetic screening technology continues to advance, the interpretation of screening results still has uncertainties. The clinical significance of many genetic variants is still unclear, especially some rare variants and newly discovered variants, which lack sufficient population data and functional validation 103. This brings challenges to clinical interpretation and counseling.

In addition, the expression of genetic variants is influenced by complex regulatory networks, such as epigenetic modifications and gene-gene interactions 104. The phenotype of the same genetic variant may not be the same in different individuals, and this incomplete penetrance makes disease risk prediction more complex 105.

Environmental factors also play an important role in the occurrence and development of diseases. Many common diseases are the result of the joint action of genetic factors and environmental factors, and genetic screening alone cannot comprehensively reflect an individual's disease risk 106. Therefore, when interpreting screening results, other factors such as family history and lifestyle need to be considered, and probabilistic risk predictions should be treated with caution.

### B. Psychological and Ethical Considerations

Genetic screening may have psychological impacts on those being screened and their family members. Positive results may cause negative emotional reactions such as anxiety and depression, especially for some incurable genetic diseases 107. Even negative results may lead to psychological problems such as survivor's guilt 108.

Informed consent is an important ethical principle of genetic screening. Those being screened have the right to know information such as the purpose, risks, and limitations of the screening, and make decisions based on their own values and preferences 109. For children and adults with impaired decision-making capacity, how to balance the benefits and potential risks of screening and ensure their best interests is an issue that needs to be carefully considered 110.

The confidentiality and privacy protection of genetic screening results are also very important. Screening results belong to personal sensitive information, and if leaked or misused, they may lead to discrimination and bias 111. In 2008, the United States passed the Genetic Information Nondiscrimination Act (GINA), prohibiting health insurance companies and employers from discriminating based on genetic information, but legal protections in areas such as life insurance and long-term care insurance are not yet perfect 112.

### C. Privacy and Discrimination Issues

With the deepening of genomic research and the rise of commercialized genetic testing, genetic privacy and discrimination issues have received increasing attention. Due to the uniqueness and sensitivity of genetic information, if improperly used or leaked, it may cause irreparable harm to individuals and families.

In the clinical field, genetic screening results may affect an individual's medical experience. Some doctors may provide differentiated treatment based on the patient's genetic information, such as reducing medical investment in "high-risk" patients 113. This gene-based discrimination not only goes against medical ethics but may also exacerbate health inequalities.

In the workplace and insurance fields, genetic discrimination issues are even more prominent. Some employers may use employees' genetic information for screening and decision-making, leading to employment discrimination 114. Insurance companies may adjust premiums or refuse coverage based on customers' genetic risks, infringing on the right to fair access to insurance 115. Although some countries have formulated relevant laws and regulations, such as the GINA Act in the United States, the implementation and supervision of these laws still face challenges in practice 116.

In addition, with the development of the Internet and big data technology, genetic privacy faces new threats. Some commercial companies may collect, use, or sell customers' genetic data without individual consent, infringing on personal privacy rights 117. Hacker attacks and data leaks may also expose individuals' genetic information to risk 118.

In summary, genetic privacy and discrimination issues are ethical and social challenges that urgently need to be resolved in the development of genetic screening. This requires joint efforts from the government, medical institutions, commercial organizations, and the public to improve laws and regulations, strengthen supervision, raise awareness throughout society, and strive to protect individual rights and interests while fully exerting the utility of genetic screening, promoting medical fairness and social harmony.

## VI. How to Choose Appropriate Genetic Screening

### A. Consult Medical Professionals

Faced with a dazzling array of genetic screening products on the market, consumers may find it difficult to make informed choices. Therefore, it is crucial to consult medical professionals before undergoing genetic screening. Doctors, genetic counselors, and other professionals can assess the necessity and suitability of screening based on an individual's and family's health history, and provide authoritative opinions on the risks, limitations, and other aspects of screening 119.

In addition, professionals can also help interpret screening results and provide personalized health guidance. Although some commercialized screenings claim to provide reports directly to consumers, the accuracy and reliability of these reports are questionable. In contrast, conducting screening and interpretation under the guidance of professionals can maximize the utility of screening and avoid unnecessary anxiety and misguidance 120.

### B. Choose Reliable Screening Institutions

Choosing a reliable screening institution is key to ensuring screening quality and personal privacy. Consumers should prioritize medical institutions or professional screening companies that have been certified by authoritative bodies and have a good reputation. In the United States, clinical-grade genetic testing needs to be approved by the U.S. Food and Drug Administration (FDA) and conducted in qualified clinical laboratories 121.

Consumers should also pay attention to the privacy protection policies of screening institutions. Reliable institutions should adopt strict data security measures and commit to not selling or sharing customers' genetic information to third parties without individual consent 122. In addition, whether the screening institution provides adequate informed consent processes and genetic counseling services is also an important indicator of its professionalism and sense of responsibility.

### C. Understand the Purpose and Limitations of Screening

Different genetic screening services have different purposes and principles. Consumers should fully understand the specific use, testing scope, and scientific basis of the screening before making a choice. Some commercialized "health risk assessment" and "dietary guidance" services may lack sufficient clinical validation, and there may be a gap between their advertised effects and actual efficacy 123.

In addition, consumers should also recognize that genetic screening is not a panacea. Many diseases are the result of the joint action of genetic factors and environmental factors, and genetic risk assessment alone cannot provide exact predictions 124. Even confirmatory genetic testing may have the possibility of false negatives or false positives. Therefore, treating genetic screening results as a reference basis rather than a determinant of health management, and maintaining a prudent attitude towards screening results, is the key to rationally treating genetic screening.

### D. Pay Attention to Relevant Laws and Regulations

Before conducting genetic screening, it is crucial to understand the relevant laws and regulations. Different countries and regions may have different regulations on genetic screening, mainly involving the following aspects:

1. Informed Consent: Most countries require obtaining informed consent from subjects before conducting genetic screening 125. This means that medical institutions need to explain in detail to the subjects the purpose, process, possible results, and their implications of the screening.

2. Data Protection: Genetic data is highly sensitive personal information, and many countries have strict data protection regulations 126. For example, the Genetic Information Nondiscrimination Act (GINA) in the United States prohibits health insurance companies and employers from discriminating based on genetic information 127.

3. Genetic Screening for Children: Many countries have special regulations for genetic screening of minors 128. Generally, it is considered appropriate only when the screening results have a direct impact on the child's health.

4. Quality Control: Some countries require institutions providing genetic screening services to obtain specific certifications or licenses 129. For example, in the United States, clinical genetic testing needs to be approved by the Food and Drug Administration (FDA) 130.

5. Result Reporting: Some countries have specific regulations on how to report genetic screening results to subjects, especially for results that may bring psychological stress 131.

When choosing genetic screening services, consumers should ensure that service providers comply with all relevant laws and regulations. At the same time, policymakers also need to continuously update regulations to adapt to the rapid development of genetic screening technology 132.

## VII. Application of Genetic Screening Results

### A. Lifestyle Adjustments

An important application of genetic screening is to guide individuals in adjusting their lifestyles based on their own genetic characteristics to prevent diseases and promote health. For some common chronic diseases such as cardiovascular disease and diabetes, early identification of genetic risk factors can help high-risk populations take preventive measures in a timely manner, such as controlling diet, increasing exercise, quitting smoking, and limiting alcohol consumption 133.

However, lifestyle adjustments need to be based on scientific evidence and vary from person to person. The impact of different genetic variants on disease risk varies, and many diseases are the result of the joint action of multiple genes and environmental factors 134. Therefore, when developing individualized lifestyle plans, both personal genetic background and other clinical and environmental risk factors need to be considered, and adjustments should be made under the guidance of professionals.

### B. Regular Check-ups and Monitoring

For certain genetic diseases or individuals carrying high-risk genetic variants, regular check-ups and monitoring are key measures for prevention and early intervention. For example, for BRCA1/2 gene mutation carriers, regular breast X-ray examinations, breast magnetic resonance imaging (MRI), and transvaginal ultrasound examinations can detect breast and ovarian cancers early and improve treatment outcomes 135.

However, formulating a reasonable check-up and monitoring plan also requires weighing the pros and cons. Overly frequent screenings may bring physical and mental burdens and medical costs, while overly long check-up intervals may miss the opportunity for early treatment 136. Therefore, individualized check-up plans should be developed based on the specific situation of the individual, on the basis of professional guidelines, and should be regularly evaluated and adjusted.

### C. Preventive Treatments or Surgeries

For certain high-risk populations, preventive treatments or surgeries can greatly reduce the incidence and mortality of specific diseases. For example, for Lynch syndrome patients, prophylactic colorectal resection can significantly reduce the risk of colorectal cancer 137. For BRCA1/2 gene mutation carriers, prophylactic bilateral mastectomy and salpingo-oophorectomy can reduce the risk of breast and ovarian cancers 138.

However, preventive treatments or surgeries are usually invasive and may have a negative impact on quality of life, as well as the risk of complications. Therefore, before making such a major medical decision, the benefits and risks of surgery need to be fully weighed, and the patient's informed consent rights must be respected 139. Adequate communication and joint decision-making between doctors and patients, as well as collaboration of multidisciplinary teams, are crucial for developing the optimal plan.

### D. Communication and Support among Family Members

The results of genetic screening are not only relevant to the individual but also closely related to the health of family members. One person's test results may imply that their blood relatives also face similar genetic risks. Therefore, sharing screening results with family members and discussing follow-up measures are important for family health management 140.

However, sharing genetic information can have complex effects on family relationships. Some people may not want to know their genetic risks, and the disclosure of screening results may bring anxiety, depression, and other psychological problems 141. Therefore, how to promote communication and mutual support among family members while respecting privacy and autonomy is an issue that needs to be treated with caution. This needs to be a key focus and guidance in the genetic counseling process.

## VIII. Conclusion

### A. The Role of Genetic Screening in Personal Health Management

In summary, genetic screening has played an increasingly important role in personal health management. It provides unprecedented opportunities for disease prediction, early diagnosis, personalized prevention, and treatment 47. By analyzing an individual's genetic information, genetic screening can help us more accurately assess health risks, develop tailored health management plans, and achieve a "prevention-oriented, precision-implemented" health management model 142.

However, genetic screening is not a panacea. It has certain limitations and uncertainties, and brings social and ethical challenges in terms of privacy, discrimination, and other aspects 143. Therefore, while fully affirming its value, we should also rationally recognize its limitations and treat screening results with caution. Genetic screening should be viewed as a beneficial tool and reference for health management, but not over-relied upon; it should be combined with other clinical information and risk factors, and utilized under professional guidance 144.

### B. Call for the Public to Raise Awareness and Attention to Genetic Screening

With the arrival of the precision medicine era, genetic screening is bound to play an increasingly important role in disease prevention and health promotion 145. As workers in the medical and public health fields, we should actively promote knowledge related to genetic screening and raise the public's awareness and participation. At the same time, we should also strengthen communication with patients and the public, listen to their voices, understand their concerns and expectations, and jointly create a good social and ecological environment 146.

As ordinary citizens, we should take the initiative to learn popular science knowledge about genetic screening and understand the value and limitations of this technology. Under the guidance of professionals and based on our own situation, we should rationally choose appropriate screening items 147. We should also face up to the psychological impact that screening may bring, learn to communicate with family members, seek social support, and jointly cope with problems and challenges that may arise.

### C. Future Development Directions of Genetic Screening

1. Popularization of Whole Genome Sequencing Technology:

- As sequencing costs continue to decrease, whole genome sequencing is expected to become a routine screening method 148.

- This will provide more comprehensive genetic information, helping to discover rare variants and complex gene interactions 149.

2. Application of Artificial Intelligence and Machine Learning:

- AI technology will help interpret large amounts of genetic data, improving the accuracy and interpretability of results 150.

- Machine learning algorithms can integrate multiple data sources to provide more precise disease risk predictions 151.

3. Development of Liquid Biopsy Technology:

- By analyzing circulating tumor DNA in blood, earlier and more convenient cancer screening may be possible 152.

- This non-invasive technology could revolutionize early diagnosis and monitoring of cancer 153.

4. Integration of Epigenomics:

- In addition to gene sequences, epigenetic modifications will also be included in screening 154.

- This will provide a more comprehensive genetic risk assessment, especially for some complex diseases 155.

5. Personalized Application of Pharmacogenomics:

- Drug response predictions based on personal genomic information will become more precise 156.

- This will further promote personalized medication, improving treatment efficacy and reducing adverse reactions 157.

6. Multi-omics Integrated Analysis:

- Combining genomics with transcriptomics, proteomics, and other multi-omics data for analysis 158.

- This systems biology approach will provide a more comprehensive health status assessment 159.

7. Application of Gene Editing Technology:

- Gene editing technologies such as CRISPR may be used to correct certain genetic defects 160.

- This brings new hope for the treatment of certain genetic diseases, but also faces ethical and safety challenges 161.

8. Combination of Wearable Devices and Genetic Data:

- Combining physiological data collected by wearable devices with genetic information to provide real-time health monitoring and risk assessment 162.

- This will promote a more proactive and continuous health management model 163.

9. Development of Community Genomics:

- Extending genetic screening to a wider population, especially in resource-limited areas 164.

- This will help reduce health inequalities and improve global disease prevention capabilities 165.

10. Improvement of Legal and Ethical Frameworks:

- As technology develops, relevant legal and ethical norms also need to be constantly updated 166.

- This will involve improvements in multiple aspects such as privacy protection, data sharing, and informed consent 167.

These development directions will jointly promote genetic screening towards more precise, comprehensive, and personalized directions, bringing revolutionary changes to future healthcare 47.

### D. Impact of Emerging Technologies on Genetic Screening

The field of genetic screening is rapidly evolving, driven by technological advancements that promise to revolutionize how we detect, interpret, and utilize genetic information. These emerging technologies are set to enhance the precision, scope, and accessibility of genetic screening, potentially transforming healthcare and personalized medicine.

1. AI-Driven Predictive Models:

- Machine learning algorithms can integrate genomic data with clinical and environmental factors to provide more accurate disease risk predictions 150.

- Deep learning models have the potential to identify complex gene-environment interaction patterns, improving our understanding of disease mechanisms 151.

- AI can aid in interpreting the functional significance of genetic variants, enhancing the clinical utility of screening results 168.

2. Integrative Genomics:

- Combining multi-omics data (genomics, transcriptomics, proteomics, etc.) provides a more comprehensive health assessment 158.

- This approach can help elucidate gene-gene interactions and epigenetic regulation, offering insights into complex diseases 158.

- Integrative genomics has the potential to identify novel biomarkers and therapeutic targets 169.

3. Single-Cell Sequencing Technology:

- Allows analysis of gene expression and variation at the single-cell level, particularly valuable for understanding tumor heterogeneity 170.

- Has the potential to revolutionize prenatal diagnosis and early cancer detection by providing high-resolution genetic information 160.

4. Gene Editing Technology:

- CRISPR and other gene editing technologies may be used to correct certain genetic defects identified through screening 171.

- This opens up possibilities for gene therapy as an intervention following genetic screening 172.

- However, it also raises significant ethical considerations that need careful examination 162.

5. Real-Time Monitoring and Wearable Devices:

- Combining genetic data with real-time physiological parameters from wearable devices could provide dynamic health risk assessments 163.

- This integration may promote a more proactive and personalized approach to preventive medicine.

6. Blockchain Technology:

- Offers a secure, decentralized method for storing and sharing genetic data 173.

- Could address issues of genetic data privacy, ownership, and consent management 174.

7. Quantum Computing:

- May significantly increase the speed and complexity of genetic data analysis in the future 175.

- Has the potential to solve current computational bottlenecks, such as protein folding prediction, which could enhance our understanding of genetic variants 176.

While these emerging technologies offer exciting possibilities, they also present new challenges. Issues of data privacy, ethical use of genetic information, and equitable access to these advanced screening methods need to be carefully addressed 177. Moreover, the integration of these technologies into clinical practice will require substantial changes in healthcare infrastructure, professional training, and public education 47.

As we move forward, it will be crucial for researchers, clinicians, policymakers, and ethicists to work collaboratively to ensure that these powerful technologies are used responsibly and effectively. By doing so, we can harness the full potential of these advancements to improve health outcomes and push the boundaries of personalized medicine 178.

For a summary of key literature in the field of genetic screening, please refer to **Table 3**. This table provides an overview of 20 seminal papers, including their main conclusions, limitations, and author notes, which together offer a comprehensive picture of the current state and future directions of genetic screening research and applications.

The importance of this review lies not only in its systematic summary of the current status of genetic screening but also in its in-depth exploration of its application prospects in personalized medicine and public health. In the current context of rapid development of precision medicine, genetic screening is becoming an important tool for disease prevention and health management.

From a clinical perspective, the significance of this study is mainly reflected in the following aspects:

1. It provides a comprehensive knowledge framework of genetic screening for clinicians, helping them better understand and apply this technology.

2. It discusses the interpretation and application of genetic screening results, which is crucial for formulating personalized prevention and treatment plans.

3. It analyzes the application of genetic screening in different disease areas, providing references for clinical practice.

4. It explores the ethical and social issues of genetic screening, helping clinicians better handle related issues in practice.

In summary, this study provides important theoretical basis and practical guidance for the application of genetic screening in clinical and public health fields.

## Figures and Tables

**Table 1:** Development History of Genetic Screening

Period	Major Developments	Impact
1960s	Robert Guthrie develops phenylketonuria screening method	Initiated newborn screening
1970s	Discovery of DNA molecular hybridization and restriction endonucleases	Laid technical foundation for genetic screening
1980s	Invention of PCR technology	Revolutionized screening efficiency
2000s	Microarray technology and next-generation sequencing technology	Large-scale genetic disease screening and improved economy

**Table 2:** Types of Genetic Screening and Their Applications

Screening Type	Main Applications	Advantages	Limitations
Prenatal Genetic Screening	Identify fetal genetic diseases or chromosomal abnormalities	Early detection of health problems	Possibility of false positives/negatives, psychological and ethical issues
Newborn Genetic Screening	Detect and treat newborn genetic diseases	Timely diagnosis, prevent adverse outcomes	Difficult to interpret results, ethical issues
Adult Disease Risk Screening	Assess adult disease susceptibility	Formulate preventive measures	Complex results, psychological impact
Cancer Genetic Screening	Early detection of hereditary tumors	Improve prognosis	Uncertain results, psychological burden
Pharmacogenetic Screening	Personalized medication	Improve efficacy, reduce adverse reactions	Challenges in genotype interpretation

**Table 3:** Summary of Key Literature in Genetic Screening

Ref. No.	Article	Conclusion	Limitations	Author Notes
12	Katsanis SH, Katsanis N. (2013) Molecular genetic testing and the future of clinical genomics	Reviews the state of molecular genetic testing and future directions	Some predictions may not have materialized	Provides a comprehensive overview of genetic testing
16	Evans JP, et al. (2013) We screen newborns, don't we?: realizing the promise of public health genomics	Argues for expanded use of genomic screening in public health	May underestimate implementation challenges	Provides a vision for genomic public health
29	Manolio TA, et al. (2009) Finding the missing heritability of complex diseases	Discusses potential sources of unexplained heritability in complex diseases	Some hypotheses remain unproven	Landmark paper in understanding complex disease genetics
31	Biesecker LG, Green RC. (2014) Diagnostic clinical genome and exome sequencing	Reviews the clinical applications of genome and exome sequencing	Rapid technological advances may outdate some information	Provides a comprehensive overview of sequencing technologies
47	Stark Z, et al. (2019) Integrating Genomics into Healthcare: A Global Responsibility	Emphasizes the need for global cooperation in implementing genomic medicine	Focuses mainly on developed countries	Highlights the importance of equity in genomic medicine
83	Relling MV, et al. (2019) Clinical Pharmacogenetics Implementation Consortium Guideline for Thiopurine Dosing Based on TPMT and NUDT15 Genotypes: 2018 Update	Provides guidelines for pharmacogenetic testing	Limited to specific genes and drugs	Important resource for clinical pharmacogenetics
105	Forrest IS, et al. (2022) Population-Based Penetrance of Deleterious Clinical Variants	Demonstrates variable penetrance of pathogenic variants across populations	Based on data from specific populations	Highlights the importance of diverse genomic databases
106	Horton RH, Lucassen AM. (2019) Recent developments in genetic/genomic medicine	Reviews recent advances in genomic medicine and their clinical implications	Rapid pace of developments may outdate some information	Provides a comprehensive overview of the field
109	Riley BD, et al. (2012) Essential elements of genetic cancer risk assessment, counseling, and testing	Provides guidelines for genetic counseling in cancer risk assessment	May not fully address newer technologies	Important resource for genetic counselors
111	Green RC, et al. (2015) GINA, genetic discrimination, and genomic medicine	Discusses the impact of the Genetic Information Nondiscrimination Act	Focuses on US policy	Highlights ongoing challenges in genetic discrimination
118	McGuire AL, et al. (2008) Confidentiality, privacy, and security of genetic and genomic test information in electronic health records	Discusses challenges in managing genetic information in electronic health records	Focuses mainly on US healthcare system	Highlights important privacy concerns
131	Appelbaum PS, et al. (2014) Models of consent to return of incidental findings in genomic research	Proposes different models for managing incidental findings in genomic research	Does not provide a definitive solution	Highlights the complexity of ethical issues in genomic research
142	Manickam K, et al. (2018) Exome Sequencing-Based Screening for BRCA1/2 Expected Pathogenic Variants Among Adult Biobank Participants	Population-based genomic screening can identify individuals with pathogenic BRCA1/2 variants	Limited to BRCA1/2 genes	Suggests potential for broader genomic screening
145	Aronson SJ, Rehm HL. (2015) Building the foundation for genomics in precision medicine	Discusses the challenges and opportunities in implementing genomic medicine	Focuses more on technical aspects than ethical considerations	Provides a roadmap for genomic medicine implementation
148	Shendure J, et al. (2017) DNA sequencing at 40: past, present and future	Reviews the history and future directions of DNA sequencing	Some future predictions may not materialize	Comprehensive overview of sequencing technology
160	Doudna JA, Charpentier E. (2014) Genome editing. The new frontier of genome engineering with CRISPR-Cas9	Introduces CRISPR- Cas9 technology and its potential applications	Does not fully address ethical concerns	Landmark paper on CRISPR technology
164	Popejoy AB, Fullerton SM. (2016) Genomics is failing on diversity	Highlights the lack of diversity in genomic studies	Focuses mainly on GWAS studies	Call to action for more inclusive genomic research
165	Landry LG, et al. (2018) Lack Of Diversity In Genomic Databases Is A Barrier To Translating Precision Medicine Research Into Practice	Highlights the importance of diversity in genomic databases	Focuses mainly on US populations	Calls for more inclusive genomic research
166	Clayton EW, et al. (2019) The law of genetic privacy: applications, implications, and limitations	Discusses legal aspects of genetic privacy	Focuses mainly on US law	Highlights the need for updated legal frameworks
167	Shabani M, Borry P. (2018) Rules for processing genetic data for research purposes in view of the new EU General Data Protection Regulation	Discusses implications of GDPR for genetic research	Focuses on EU regulations	Important for researchers working with EU data

## References

[1] Comabella M, Sastre-Garriga J, Montalban X (2016). Precision medicine in multiple sclerosis: biomarkers for diagnosis, prognosis, and treatment response. Curr Opin Neurol.

[2] Schwartzberg L, Kim ES, Liu D, Schrag D (2017). Precision Oncology: Who, How, What, When, and When Not?. Am Soc Clin Oncol Educ Book.

[3] Musunuru K, Hershberger RE, Day SM (2020). Genetic Testing for Inherited Cardiovascular Diseases: A Scientific Statement From the American Heart Association. Circ Genom Precis Med.

[4] Prior-de Castro C, Gómez-González C, Rodríguez-López R, Macher HC; Prenatal Diagnosis Commission and the Genetics Commission of the Spanish Society of Laboratory Medicine (2023). Prenatal genetic diagnosis of monogenic diseases. Adv Lab Med.

[5] Wolf CM (2019). Hypertrophic cardiomyopathy: genetics and clinical perspectives. Cardiovasc Diagn Ther.

[6] Feero WG, Guttmacher AE, Collins FS (2010). Genomic medicine-an updated primer. N Engl J Med.

[7] Kim N, Kong SY, Yoo J, Kim DH, Seo SH, Kim J (2022). Current Issues, Challenges, and Future Per-spectives of Genetic Counseling in Korea. Ann Lab Med.

[8] Chien YH, Hwu WL, Lee NC (2019). Newborn screening: Taiwanese experience. Ann Transl Med.

[9] Zhang L, Bao Y, Riaz M (2019). Population genomic screening of all young adults in a health-care system: a cost-effectiveness analysis. [Published correction appears in Genet Med. 2019 Apr 4;:]. Genet Med.

[10] Prince AE, Roche MI (2014). Genetic information, non-discrimination, and privacy protections in genetic counseling practice. J Genet Couns.

[11] Henneman L, Vermeulen E, van El CG, Claassen L, Timmermans DR, Cornel MC (2013). Public attitudes towards genetic testing revisited: comparing opinions between 2002 and 2010. Eur J Hum Genet.

[12] Katsanis SH, Katsanis N (2013). Molecular genetic testing and the future of clinical genomics. Nat Rev Genet.

[13] Chen HF, Chen SU, Ma GC (2018). Preimplantation genetic diagnosis and screening: Current status and future challenges. J Formos Med Assoc.

[14] Burke W (2014). Genetic tests: clinical validity and clinical utility. Curr Protoc Hum Genet.

[15] Ardekani AM (2009). Genetic technologies and ethics. J Med Ethics Hist Med.

[16] Evans JP, Berg JS, Olshan AF, Magnuson T, Rimer BK (2013). We screen newborns, don't we?: realizing the promise of public health genomics. Genet Med.

[17] Hung TH Challenges and strategies of international cooperation in Taiwan biobanks - centered on legal and ethical issues [master's thesis, National Tsing Hua University]. 2015.

[18] Karki R, Pandya D, Elston RC, Ferlini C (2015). Defining "mutation" and "polymorphism" in the era of personal genomics. BMC Med Genomics.

[19] Cooper DN, Krawczak M, Polychronakos C, Tyler-Smith C, Kehrer-Sawatzki H (2013). Where genotype is not predictive of phenotype: towards an understanding of the molecular basis of reduced penetrance in human inherited disease. Hum Genet.

[20] Bartlett JMS, Stirling D (2003). A Short History of the Polymerase Chain Reaction. PCR Protocols. Methods in Molecular Biology 226 2nd.

[21] Heather JM, Chain B (2016). The sequence of sequencers: The history of sequencing DNA. Genomics.

[22] LaFramboise T (2009). Single nucleotide polymorphism arrays: a decade of biological, computational and technological advances. Nucleic Acids Res.

[23] Mardis ER (2017). DNA sequencing technologies: 2006-2016. Nat Protoc.

[24] Nussbaum RL, McInnes RR, Willard HF (2016). Thompson & Thompson Genetics in Medicine. 8th ed. Philadelphia: Elsevier.

[25] Hassold T, Hunt P (2001). To err (meiotically) is human: the genesis of human aneuploidy. Nat Rev Genet.

[26] Karczewski KJ, Francioli LC, Tiao G (2021). The mutational constraint spectrum quantified from variation in 141,456 humans. [Published correction appears in Nature].

[27] Carvill GL, Matheny T, Hesselberth J, Demarest S (2021). Haploinsufficiency, Dominant Negative, and Gain-of-Function Mechanisms in Epilepsy: Matching Therapeutic Approach to the Pathophysiology. Neurotherapeutics.

[28] Strachan T, Read A (2018). Human Molecular Genetics. 5th ed. New York: Garland Science.

[29] Manolio TA, Collins FS, Cox NJ (2009). Finding the missing heritability of complex diseases. Nature.

[30] Feinberg AP (2018). The Key Role of Epigenetics in Human Disease Prevention and Mitigation. N Engl J Med.

[31] Biesecker LG, Green RC (2014). Diagnostic clinical genome and exome sequencing. N Engl J Med.

[32] Mullis K, Faloona F, Scharf S, Saiki R, Horn G, Erlich H (1986). Specific enzymatic amplification of DNA in vitro: the polymerase chain reaction. Cold Spring Harb Symp Quant Biol.

[33] Kerem B, Rommens JM, Buchanan JA (1989). Identification of the cystic fibrosis gene: genetic analysis. Science.

[34] Garibyan L, Avashia N (2013). Polymerase chain reaction. J Invest Dermatol.

[35] Sanger F, Nicklen S, Coulson AR (1977). DNA sequencing with chain-terminating inhibitors. Proc Natl Acad Sci U S A.

[36] Shendure J, Ji H (2008). Next-generation DNA sequencing. Nat Biotechnol.

[37] Goodwin S, McPherson JD, McCombie WR (2016). Coming of age: ten years of next-generation sequencing technologies. Nat Rev Genet.

[38] Metzker ML (2010). Sequencing technologies - the next generation. Nat Rev Genet.

[39] Bumgarner R (2013). Overview of DNA microarrays: types, applications, and their future. Curr Protoc Mol Biol.

[40] LaFramboise T (2009). Single nucleotide polymorphism arrays: a decade of biological, computational and technological advances. Nucleic Acids Res.

[41] Vogelstein B, Kinzler KW (1999). Digital PCR. Proc Natl Acad Sci U S A.

[42] Hindson CM, Chevillet JR, Briggs HA (2013). Absolute quantification by droplet digital PCR versus analog real-time PCR. Nat Methods.

[43] Eid J, Fehr A, Gray J (2009). Real-time DNA sequencing from single polymerase molecules. Science.

[44] Sedlazeck FJ, Lee H, Darby CA, Schatz MC (2018). Piercing the dark matter: bioinformatics of long-range sequencing and mapping. Nat Rev Genet.

[45] Laird PW (2010). Principles and challenges of genomewide DNA methylation analysis. Nat Rev Genet.

[46] Rivera CM, Ren B (2013). Mapping human epigenomes. Cell.

[47] Stark Z, Dolman L, Manolio TA (2019). Integrating Genomics into Healthcare: A Global Responsibility. Am J Hum Genet.

[48] GUTHRIE R, SUSI A (1963). A SIMPLE PHENYLALANINE METHOD FOR DETECTING PHENYLKETONURIA IN LARGE POPULATIONS OF NEWBORN INFANTS. Pediatrics.

[49] Southern EM (1975). Detection of specific sequences among DNA fragments separated by gel electrophoresis. J Mol Biol.

[50] Hutchison CA 3rd (2007). DNA sequencing: bench to bedside and beyond. Nucleic Acids Res.

[51] Saiki RK, Gelfand DH, Stoffel S (1988). Primer-directed enzymatic amplification of DNA with a thermostable DNA polymerase. Science.

[52] McPherson E (2006). Genetic diagnosis and testing in clinical practice. Clin Med Res.

[53] Hacia JG, Fan JB, Ryder O (1999). Determination of ancestral alleles for human single-nucleotide polymorphisms using high-density oligonucleotide arrays. Nat Genet.

[54] Bentley DR, Balasubramanian S, Swerdlow HP (2008). Accurate whole human genome sequencing using reversible terminator chemistry. Nature.

[55] Rothberg JM, Hinz W, Rearick TM (2011). An integrated semiconductor device enabling non-optical genome sequencing. Nature.

[56] Alldred SK, Takwoingi Y, Guo B (2017). First and second trimester serum tests with and without first trimester ultrasound tests for Down's syndrome screening. Cochrane Database Syst Rev.

[57] Fesahat F, Montazeri F, Hoseini SM (2020). Preimplantation genetic testing in assisted reproduction technology. J Gynecol Obstet Hum Reprod.

[58] Gil MM, Accurti V, Santacruz B, Plana MN, Nicolaides KH (2017). Analysis of cell-free DNA in maternal blood in screening for aneuploidies: updated meta-analysis. Ultrasound Obstet Gynecol.

[59] Gekas J, Langlois S, Ravitsky V (2016). Non-invasive prenatal testing for fetal chromosome abnormalities: review of clinical and ethical issues. Appl Clin Genet.

[60] Dondorp W, de Wert G, Bombard Y (2015). Non-invasive prenatal testing for aneuploidy and beyond: challenges of responsible innovation in prenatal screening. [Published correction appears in Eur J Hum Genet. 2015 Nov;23(11):1592]. Eur J Hum Genet.

[61] Dondorp W, de Wert G (2019). Refining the ethics of preimplantation genetic diagnosis: A plea for contextualized proportionality. Bioethics.

[62] Bunnik EM, de Jong A, Nijsingh N, de Wert GM (2013). The new genetics and informed consent: differentiating choice to preserve autonomy. Bioethics.

[63] Pitt JJ (2010). Newborn screening. Clin Biochem Rev.

[64] Chan MJ, Liao HC, Gelb MH (2019). Taiwan National Newborn Screening Program by Tandem Mass Spectrometry for Mucopolysaccharidoses Types I, II, and VI. J Pediatr.

[65] Vockley J, Andersson HC, Antshel KM (2014). Phenylalanine hydroxylase deficiency: diagnosis and management guideline. [Published correction appears in Genet Med. 2014 Apr;16(4):356]. Genet Med.

[66] LaFranchi SH (2011). Approach to the diagnosis and treatment of neonatal hypothyroidism. J Clin Endocrinol Metab.

[67] Tluczek A, Orland KM, Cavanagh L (2011). Psychosocial consequences of false-positive newborn screens for cystic fibrosis. Qual Health Res.

[68] Botkin JR, Goldenberg AJ, Rothwell E, Anderson RA, Lewis MH (2013). Retention and research use of residual newborn screening bloodspots. Pediatrics.

[69] Torkamani A, Wineinger NE, Topol EJ (2018). The personal and clinical utility of polygenic risk scores. Nat Rev Genet.

[70] Kuchenbaecker KB, Hopper JL, Barnes DR (2017). Risks of Breast, Ovarian, and Contralateral Breast Cancer for BRCA1 and BRCA2 Mutation Carriers. JAMA.

[71] Rabinovici GD (2021). Controversy and Progress in Alzheimer's Disease - FDA Approval of Aducanumab. N Engl J Med.

[72] Lewis CM, Vassos E (2020). Polygenic risk scores: from research tools to clinical instruments. Genome Med.

[73] Chasioti D, Yan J, Nho K, Saykin AJ (2019). Progress in Polygenic Composite Scores in Alzheimer's and Other Complex Diseases. Trends Genet.

[74] Oliveri S, Ferrari F, Manfrinati A, Pravettoni G (2018). A Systematic Review of the Psychological Implications of Genetic Testing: A Comparative Analysis Among Cardiovascular, Neurodegenerative and Cancer Diseases. Front Genet.

[75] Desmond A, Kurian AW, Gabree M (2015). Clinical Actionability of Multigene Panel Testing for Hereditary Breast and Ovarian Cancer Risk Assessment. JAMA Oncol.

[76] Lorans M, Dow E, Macrae FA, Winship IM, Buchanan DD (2018). Update on Hereditary Colorectal Cancer: Improving the Clinical Utility of Multigene Panel Testing. Clin Colorectal Cancer.

[77] Yurgelun MB, Hiller E, Garber JE (2015). Population-Wide Screening for Germline BRCA1 and BRCA2 Mutations: Too Much of a Good Thing?. J Clin Oncol.

[78] Lincoln SE, Nussbaum RL, Kurian AW (2021). Yield and Utility of Germline Testing Following Tumor Sequencing in Patients With Cancer. [Published correction appears in JAMA Netw Open].

[79] Schenberg T, Mitchell G (2014). Prophylactic bilateral salpingectomy as a prevention strategy in women at high-risk of ovarian cancer: a mini-review. Front Oncol.

[80] Lauschke VM, Milani L, Ingelman-Sundberg M (2017). Pharmacogenomic Biomarkers for Improved Drug Therapy-Recent Progress and Future Developments. AAPS J.

[81] Scott SA, Sangkuhl K, Stein CM (2013). Clinical Pharmacogenetics Implementation Consortium guidelines for CYP2C19 genotype and clopidogrel therapy: 2013 update. Clin Pharmacol Ther.

[82] Tantry US, Gesheff M, Liu F, Bliden KP, Gurbel PA (2014). Resistance to antiplatelet drugs: what progress has been made?. Expert Opin Pharmacother.

[83] Relling MV, Schwab M, Whirl-Carrillo M (2019). Clinical Pharmacogenetics Implementation Consortium Guideline for Thiopurine Dosing Based on TPMT and NUDT15 Genotypes: 2018 Update. Clin Pharmacol Ther.

[84] Rao Y, Zhao Q, Zhang X, Yang H, Lou Y, Zhang X (2016). Current status and future prospects of the development of clinical Pharmacy in China: A SWOT analysis. Pak J Pharm Sci.

[85] Roden DM, McLeod HL, Relling MV (2019). Pharmacogenomics. Lancet.

[86] Zhou Y, Peng S, Wang H, Cai X, Wang Q (2024). Review of Personalized Medicine and Pharmacogenomics of Anti-Cancer Compounds and Natural Products. Genes.

[87] Manosroi W, Williams GH (2019). Genetics of Human Primary Hypertension: Focus on Hormonal Mechanisms. Endocr Rev.

[88] Nelson HD, Pappas M, Cantor A, Haney E, Holmes R (2019). Risk Assessment, Genetic Counseling, and Genetic Testing for BRCA-Related Cancer in Women: Updated Evidence Report and Systematic Review for the US Preventive Services Task Force. JAMA.

[89] Knowles JW, Rader DJ, Khoury MJ (2017). Cascade Screening for Familial Hypercholesterolemia and the Use of Genetic Testing. JAMA.

[90] Godard B, Raeburn S, Pembrey M, Bobrow M, Farndon P, Aymé S (2003). Genetic information and testing in insurance and employment: technical, social and ethical issues. Eur J Hum Genet.

[91] Phillips KA, Veenstra DL, Oren E, Lee JK, Sadee W (2001). Potential role of pharmacogenomics in reducing adverse drug reactions: a systematic review. JAMA.

[92] Dunnenberger HM, Crews KR, Hoffman JM (2015). Preemptive clinical pharmacogenetics implementation: current programs in five US medical centers. Annu Rev Pharmacol Toxicol.

[93] Wu CT, Wang SM, Su YE (2022). A Precision Health Service for Chronic Diseases: Development and Cohort Study Using Wearable Device, Machine Learning, and Deep Learning. IEEE J Transl Eng Health Med.

[94] Blazer KR, Slavin T, Weitzel JN (2016). Increased Reach of Genetic Cancer Risk Assessment as a Tool for Precision Management of Hereditary Breast Cancer. JAMA Oncol.

[95] Manchanda R, Burnell M, Gaba F (2020). Randomised trial of population-based BRCA testing in Ashkenazi Jews: long-term outcomes. BJOG.

[96] Ross LF, Saal HM, David KL, Anderson RR; American Academy of Pediatrics; American College of Medical Genetics, Genomics (2013). Technical report: Ethical and policy issues in genetic testing and screening of children. [Published correction appears in Genet Med]. 2013 Apr;15(4):321. Ross, Laine Friedman [corrected to Ross, Lainie Friedman]]. Genet Med.

[97] Yanes T, Meiser B, Young MA (2017). Psychosocial and behavioral impact of breast cancer risk assessed by testing for common risk variants: protocol of a prospective study. BMC Cancer.

[98] Watts GF, Gidding S, Wierzbicki AS (2014). Integrated guidance on the care of familial hypercholesterolemia from the International FH Foundation. J Clin Lipidol.

[99] Metcalfe SA (2012). Carrier screening in preconception consultation in primary care. J Community Genet.

[100] Bodurtha J, Strauss JF 3rd (2012). Genomics and perinatal care. N Engl J Med.

[101] Chan JL, Johnson LNC, Sammel MD (2017). Reproductive Decision-Making in Women with BRCA1/2 Mutations. J Genet Couns.

[102] Knoers N, Antignac C, Bergmann C (2022). Genetic testing in the diagnosis of chronic kidney disease: recommendations for clinical practice. Nephrol Dial Transplant.

[103] Rehm HL, Berg JS, Brooks LD (2015). ClinGen-the Clinical Genome Resource. N Engl J Med.

[104] Maier RM, Zhu Z, Lee SH (2018). Improving genetic prediction by leveraging genetic correlations among human diseases and traits. Nat Commun.

[105] Forrest IS, Chaudhary K, Vy HMT (2022). Population-Based Penetrance of Deleterious Clinical Variants. JAMA.

[106] Horton RH, Lucassen AM (2019). Recent developments in genetic/genomic medicine. Clin Sci (Lond).

[107] Rahman B, Meiser B, Sachdev P (2012). To know or not to know: an update of the literature on the psychological and behavioral impact of genetic testing for Alzheimer disease risk. Genet Test Mol Biomarkers.

[108] Funanage VL (2021). Impact of Genetic Testing on Human Health: The Current Landscape and Future for Personalized Medicine. Dela J Public Health.

[109] Riley BD, Culver JO, Skrzynia C (2012). Essential elements of genetic cancer risk assessment, counseling, and testing: updated recommendations of the National Society of Genetic Counselors. J Genet Couns.

[110] Green RC, Goddard KAB, Jarvik GP (2016). Clinical Sequencing Exploratory Research Consortium: Accelerating Evidence-Based Practice of Genomic Medicine. [Published correction appears in Am J Hum Genet. 2016 Jul 7;99(1):246. doi: 10.1016/j.ajhg.2016.06.002]. Am J Hum Genet.

[111] Green RC, Lautenbach D, McGuire AL (2015). GINA, genetic discrimination, and genomic medicine. N Engl J Med.

[112] Yanes T, Kaur R, Meiser B (2020). Women's responses and understanding of polygenic breast cancer risk information. Fam Cancer.

[113] Joly Y, Marrocco G, Dupras C (2019). Risks of compulsory genetic databases. Science.

[114] Bélisle-Pipon JC, Vayena E, Green RC, Cohen IG (2019). Genetic testing, insurance discrimination and medical research: what the United States can learn from peer countries. Nat Med.

[115] Tiller J, Morris S, Rice T (2020). Genetic discrimination by Australian insurance companies: a survey of consumer experiences. [Published correction appears in Eur J Hum Genet].

[116] Rothstein MA, Anderlik MR (2001). What is genetic discrimination, and when and how can it be prevented?. Genet Med.

[117] Du L, Wang M (2020). Genetic Privacy and Data Protection: A Review of Chinese Direct-to-Consumer Genetic Test Services. Front Genet.

[118] McGuire AL, Fisher R, Cusenza P (2008). Confidentiality, privacy, and security of genetic and genomic test information in electronic health records: points to consider. Genet Med.

[119] Hayward J, Evans W, Miller E, Rafi I (2023). Embedding genomics across the NHS: a primary care perspective. Future Healthc J.

[120] Haggerty J, Tudiver F, Brown JB, Herbert C, Ciampi A, Guibert R (2005). Patients' anxiety and expectations: how they influence family physicians' decisions to order cancer screening tests. Can Fam Physician.

[121] Sanderson S, Zimmern R, Kroese M, Higgins J, Patch C, Emery J (2005). How can the evaluation of genetic tests be enhanced? Lessons learned from the ACCE framework and evaluating genetic tests in the United Kingdom. Genet Med.

[122] Caulfield T, Ries NM, Ray PN, Shuman C, Wilson B (2010). Direct-to-consumer genetic testing: good, bad or benign?. Clin Genet.

[123] Rini C, Khan CM, Moore E (2018). The who, what, and why of research participants' intentions to request a broad range of secondary findings in a diagnostic genomic sequencing study. Genet Med.

[124] Manrai AK, Patel CJ, Ioannidis JPA (2018). In the Era of Precision Medicine and Big Data, Who Is Normal?. JAMA.

[125] Niemiec E, Howard HC (2016). Ethical issues in consumer genome sequencing: Use of consumers' samples and data. Appl Transl Genom.

[126] Phillips AM (2016). 'Only a click away - DTC genetics for ancestry, health, love…and more: A view of the business and regulatory landscape'. Appl Transl Genom.

[127] Rothstein MA (2018). GINA at Ten and the Future of Genetic Nondiscrimination Law. Hastings Cent Rep.

[128] COMMITTEE ON BIOETHICS; COMMITTEE ON GENETICS, AND; AMERICAN COL-LEGE OF MEDICAL GENETICS AND; GENOMICS SOCIAL; ETHICAL; LEGAL ISSUES COMMITTEE (2013). Ethical and policy issues in genetic testing and screening of children. Pediat-rics.

[129] Kalokairinou L, Howard HC, Slokenberga S (2018). Legislation of direct-to-consumer genetic testing in Europe: a fragmented regulatory landscape. J Community Genet.

[130] Evans BJ, Burke W, Jarvik GP (2015). The FDA and genomic tests-getting regulation right. N Engl J Med.

[131] Appelbaum PS, Parens E, Waldman CR (2014). Models of consent to return of incidental findings in genomic research. Hastings Cent Rep.

[132] de Paor A (2014). Regulating genetic information-exploring the options in legal theory. Eur J Health Law.

[133] Mega JL, Sabatine MS, Antman EM (2014). Population and personalized medicine in the modern era. JAMA.

[134] Bauer DC, Gaff C, Dinger ME (2014). Genomics and personalised whole-of-life healthcare. Trends Mol Med.

[135] Peshkin BN, Isaacs C, Finch C, Kent S, Schwartz MD (2003). Tamoxifen as chemoprevention in BRCA1 and BRCA2 mutation carriers with breast cancer: a pilot survey of physicians. J Clin Oncol.

[136] Landi MT, Consonni D, Rotunno M (2008). Environment And Genetics in Lung cancer Etiology (EAGLE) study: an integrative population-based case-control study of lung cancer. BMC Public Health.

[137] Lynch HT, Shaw TG (2013). Practical genetics of colorectal cancer. Chin Clin Oncol.

[138] Weitzel JN, Blazer KR, MacDonald DJ, Culver JO, Offit K (2011). Genetics, genomics, and cancer risk assessment: State of the Art and Future Directions in the Era of Personalized Medicine. CA Cancer J Clin.

[139] Shah PD, Domchek SM (2020). The contemporary landscape of genetic testing and breast cancer: Emerging issues. Breast J.

[140] Jacobs C, Patch C, Michie S (2019). Communication about genetic testing with breast and ovarian cancer patients: a scoping review. Eur J Hum Genet.

[141] Mendes Á, Paneque M, Sousa L, Clarke A, Sequeiros J (2016). How communication of genetic information within the family is addressed in genetic counselling: a systematic review of research evidence. Eur J Hum Genet.

[142] Manickam K, Buchanan AH, Schwartz MLB (2018). Exome Sequencing-Based Screening for BRCA1/2 Expected Pathogenic Variants Among Adult Biobank Participants. JAMA Netw Open.

[143] Green RC, Goddard KAB, Jarvik GP (2016). Clinical Sequencing Exploratory Research Consortium: Accelerating Evidence-Based Practice of Genomic Medicine. Am J Hum Genet.

[144] Ashley EA (2015). The precision medicine initiative: a new national effort. JAMA.

[145] Aronson SJ, Rehm HL (2015). Building the foundation for genomics in precision medicine. Nature.

[146] Halverson CM, Ross LF (2012). Engaging African-Americans about biobanks and the return of research results. J Community Genet.

[147] Haga SB, Barry WT, Mills R (2013). Public knowledge of and attitudes toward genetics and genetic testing. Genet Test Mol Biomarkers.

[148] Shendure J, Balasubramanian S, Church GM (2017). DNA sequencing at 40: past, present and future. Nature.

[149] Hou Y, Guo H, Cao C (2016). Single-cell triple omics sequencing reveals genetic, epigenetic, and transcriptomic heterogeneity in hepatocellular carcinomas. Cell Res.

[150] Zou J, Huss M, Abid A, Mohammadi P, Torkamani A, Telenti A (2019). A primer on deep learning in genomics. Nat Genet.

[151] Eraslan G, Avsec Ž, Gagneur J, Theis FJ (2019). Deep learning: new computational modelling techniques for genomics. Nat Rev Genet.

[152] Wan JCM, Massie C, Garcia-Corbacho J (2017). Liquid biopsies come of age: towards implementation of circulating tumour DNA. Nat Rev Cancer.

[153] Crowley E, Di Nicolantonio F, Loupakis F, Bardelli A (2013). Liquid biopsy: monitoring cancer-genetics in the blood. Nat Rev Clin Oncol.

[154] Lappalainen T, Greally JM (2017). Associating cellular epigenetic models with human phenotypes. Nat Rev Genet.

[155] Hannon E, Knox O, Sugden K (2018). Characterizing genetic and environmental influences on variable DNA methylation using monozygotic and dizygotic twins. PLoS Genet.

[156] Relling MV, Evans WE (2015). Pharmacogenomics in the clinic. Nature.

[157] Weinshilboum RM, Wang L (2017). Pharmacogenomics: Precision Medicine and Drug Response. Mayo Clin Proc.

[158] Hasin Y, Seldin M, Lusis A (2017). Multi-omics approaches to disease. Genome Biol.

[159] Karczewski KJ, Snyder MP (2018). Integrative omics for health and disease. Nat Rev Genet.

[160] Doudna JA, Charpentier E (2014). Genome editing. The new frontier of genome engineering with CRISPR-Cas9. Science.

[161] Cai L, Fisher AL, Huang H, Xie Z (2016). CRISPR-mediated genome editing and human diseases. Genes Dis.

[162] Dunn J, Runge R, Snyder M (2018). Wearables and the medical revolution. Per Med.

[163] Li X, Dunn J, Salins D (2017). Digital Health: Tracking Physiomes and Activity Using Wearable Biosensors Reveals Useful Health-Related Information. PLoS Biol.

[164] Popejoy AB, Fullerton SM (2016). Genomics is failing on diversity. Nature.

[165] Landry LG, Ali N, Williams DR, Rehm HL, Bonham VL (2018). Lack Of Diversity In Genomic Databases Is A Barrier To Translating Precision Medicine Research Into Practice. Health Aff (Millwood).

[166] Clayton EW, Evans BJ, Hazel JW, Rothstein MA (2019). The law of genetic privacy: applications, implications, and limitations. J Law Biosci.

[167] Shabani M, Borry P (2018). Rules for processing genetic data for research purposes in view of the new EU General Data Protection Regulation. Eur J Hum Genet.

[168] Sundaram L, Gao H, Padigepati SR (2019). Predicting the clinical impact of human mutation with deep neural networks. [Published correction appears in Nat Genet].

[169] Papalexi E, Satija R (2018). Single-cell RNA sequencing to explore immune cell heterogeneity. Nat Rev Immunol.

[170] Macaulay IC, Voet T (2014). Single cell genomics: advances and future perspectives. PLoS Genet.

[171] Dunbar CE, High KA, Joung JK, Kohn DB, Ozawa K, Sadelain M (2018). Gene therapy comes of age. Science.

[172] Brokowski C, Adli M (2019). CRISPR Ethics: Moral Considerations for Applications of a Powerful Tool. J Mol Biol.

[173] Ozercan HI (2018). Realizing the potential of blockchain technologies in genomics. Genome Res.

[174] Shabani M (2019). Blockchain-based platforms for genomic data sharing: a de-centralized approach in response to the governance problems?. J Am Med Inform Assoc.

[175] Cao Y, Romero J, Olson JP (2019). Quantum Chemistry in the Age of Quantum Computing. Chem Rev.

[176] Outeiral C, Strahm M, Shi J (2021). The prospects of quantum computing in computational molecular biology. WIREs Comput Mol Sci.

[177] Braverman G, Shapiro ZE, Bernstein JA (2018). Ethical Issues in Contemporary Clinical Genetics. Mayo Clin Proc Innov Qual Outcomes.

[178] Ginsburg GS, Phillips KA (2018). Precision Medicine: From Science To Value. Health Aff (Millwood).

